# Supplementary material for: Virulence, multiple drug resistance, and biofilm-formation in Salmonella species isolated from layer, broiler, and dual-purpose indigenous chickens
Source: PLoS One. 2024 Oct 28;19(10):e0310010. doi: 10.1371/journal.pone.0310010 (PMC11515961; doi:10.1371/journal.pone.0310010)
Supplement: S1 File — (DOCX) [file pone.0310010.s001.docx]

**Interview Questions**

**Towards *Salmonella*-specific bacteriophage therapy for broiler chickens: Physiological and meat quality responses**

**.**

1. This study is meant to investigate some selected poultry farming practices in all farms that will form part of study.
2. You have been particularly selected to part-take in this survey due to your management role in the poultry farm.
3. The answers you provide will be used for the purpose of this investigation only, confidentiality will be maintained throughout.

**Name of the farm** _________________________________________________________

**Do you agree to take this survey? (Tick appropriate box)**

| **Yes** | **No** |
| --- | --- |
|  |  |

**SECTION A (GENERAL)**

1. Gender (Please tick appropriate box)

| **Male** | **Female** |
| --- | --- |
|  |  |

1. How many farm workers report to you? (State number)

__________________________________________________________________________________

1. How long have you served in this position as a farm manager/owner? (Tick appropriate box)

| **0-1 year** | **1-3 years** | **3-7 years** | **7-10 years** | **10 + years** |
| --- | --- | --- | --- | --- |
|  |  |  |  |  |

1. How many chickens do you keep per production cycle? (State number)

__________________________________________________________________________________

**SECTION B (CONTENT)**

1. Where do you get your poultry feed (source of feed)? **(Tick appropriate box)**

| **Home made** | **Retail shops** |
| --- | --- |
|  |  |

1. Do you give your poultry any medical treatment such as anthelmintics and antibiotics? **(Tick appropriate box)**

| **Yes** | **No** |
| --- | --- |
|  |  |

1. Do you use antibiotics in your farm? **(Tick appropriate box)**

| **Yes** | **No** |
| --- | --- |
|  |  |

If yes, how frequently do you use antibiotics. **(Tick appropriate box)**

| **Once per production cycle** | **Only when required** | **Most of the time** |
| --- | --- | --- |
|  |  |  |

1. Do you vaccinate your poultry against *Salmonella*? **(Tick appropriate box)**

| **Yes** | **No** |
| --- | --- |
|  |  |

1. Does wild birds have access to you facilities? **(Tick appropriate box)**

| **Yes** | **No** |
| --- | --- |
|  |  |

1. Do you or your farm workers own any other domestic animals (chickens)? (Tick appropriate box)

| **Dogs** | **Cats** | **Ducks** | **chickens** | **Birds** |
| --- | --- | --- | --- | --- |
|  |  |  |  |  |

**Other (specify)**

1. Which biosecurity measures are in place? (Tick all applicable answers)

| **Foot bath** | **Car disinfectants** | **Uniform** | **Cleaning of equipment’s and facilities** | **Regulation of visitors** |
| --- | --- | --- | --- | --- |
|  |  |  |  |  |

**Other (specify)**

**SECTION C (CLOSURE)**

1. What is your general comment regarding the survey?

__________________________________________________________________________________

__________________________________________________________________________________

__________________________________________________________________________________

__________________________________________________________________________________

*****END OF SURVEY*****
