# Supplementary material for: Virulence, multiple drug resistance, and biofilm-formation in Salmonella species isolated from layer, broiler, and dual-purpose indigenous chickens
Source: PLoS One. 2024 Oct 28;19(10):e0310010. doi: 10.1371/journal.pone.0310010 (PMC11515961; doi:10.1371/journal.pone.0310010)
Supplement: S2 File — (DOCX) [file pone.0310010.s002.docx]

**Supplementary material 2:** Poultry farms husbandry practices and their characteristics

| **Characteristics** | **Frequency (%)** |
| --- | --- |
| **Gender** |  |
| Male | 9(60) |
| Female | 6(40) |
| **No of Employees** |  |
| 1 to 5 | 11(79) |
| 6 to 10 | 2(14) |
| 11 and above | 1(7) |
| **Farming experience** | |
| 1 to 3 | 5(33) |
| 4 to 7 | 3(20) |
| 8 to 10 | 2(13) |
| 10 and above | 5(33) |
| **Flock size** |  |
| 0 -2000 | 11(73) |
| 2001 – 5000 | 2(13) |
| 5001 and above | 2(13) |
| **Feed source** |  |
| Retail | 10(67) |
| Home made | 5(33) |
| **Use of treatment** |  |
| No | 7(47) |
| Yes | 8(53) |
| **Antibiotic use** |  |
| No | 5(33) |
| Yes | 10(67) |
| **Knowledge of Salmonella** | |
| No | 9(60) |
| Yes | 6(40) |
| **Wild birds access** |  |
| No | 8(53) |
| Yes | 7(47) |
| **Biosecurity** |  |
| None | 9(60) |
| Footbath and uniform | 2(13) |
| Cars and human disinfection plus uniform | 4(27) |
