# Supplementary material for: Virulence, multiple drug resistance, and biofilm-formation in Salmonella species isolated from layer, broiler, and dual-purpose indigenous chickens
Source: PLoS One. 2024 Oct 28;19(10):e0310010. doi: 10.1371/journal.pone.0310010 (PMC11515961; doi:10.1371/journal.pone.0310010)
Supplement: S1 Raw images — (PDF) [file pone.0310010.s003.pdf]

The original uncropped and unadjusted images underlying all gel results reported in the manuscript.

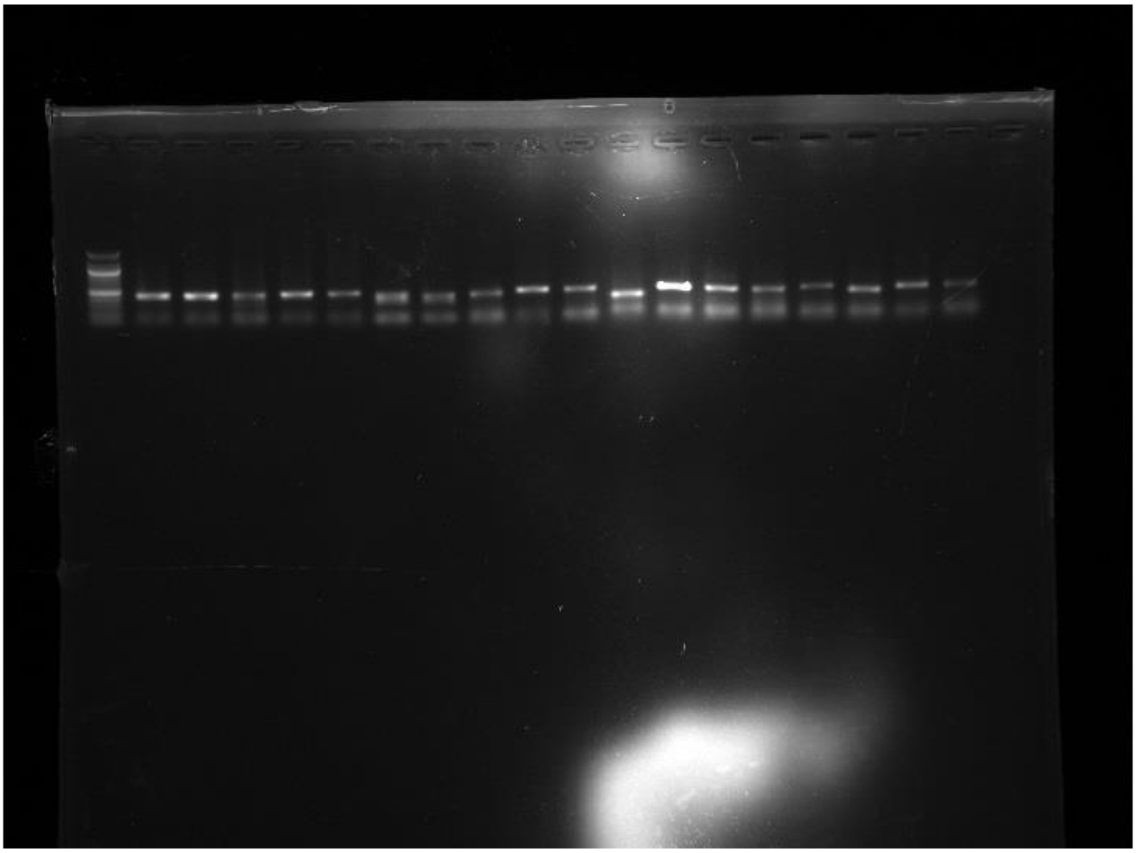

Fig 1: Agarose gel electrophoresis depicting *Salmonella species* specific *invA* gene from confirmed isolates.

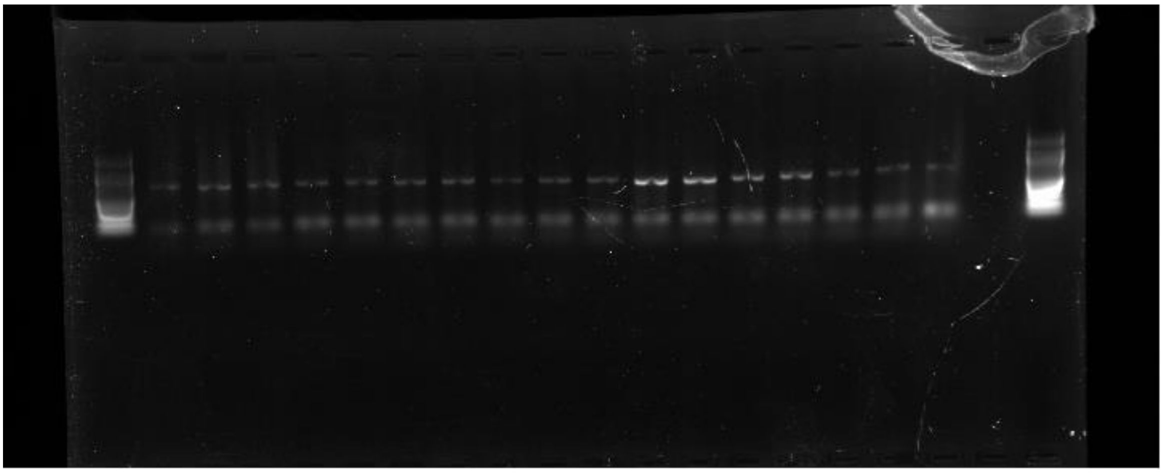

Fig 2: Agarose gel electrophoresis depicting *Salmonella species* specific *fliC* gene from confirmed isolates.

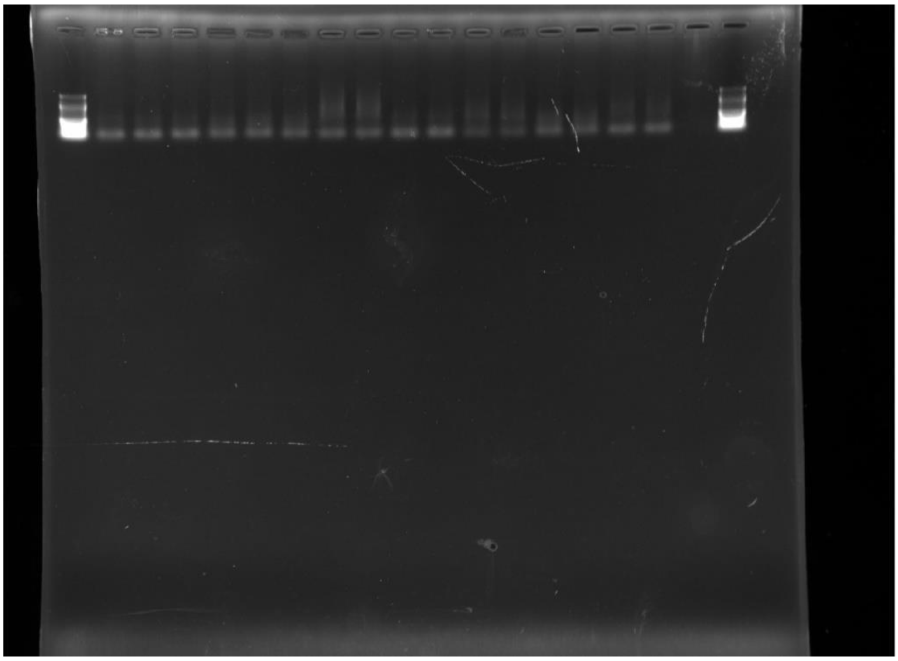

Fig 3: Agarose gel electrophoresis depicting *Salmonella species* specific *Prot6e* gene from confirmed isolates.

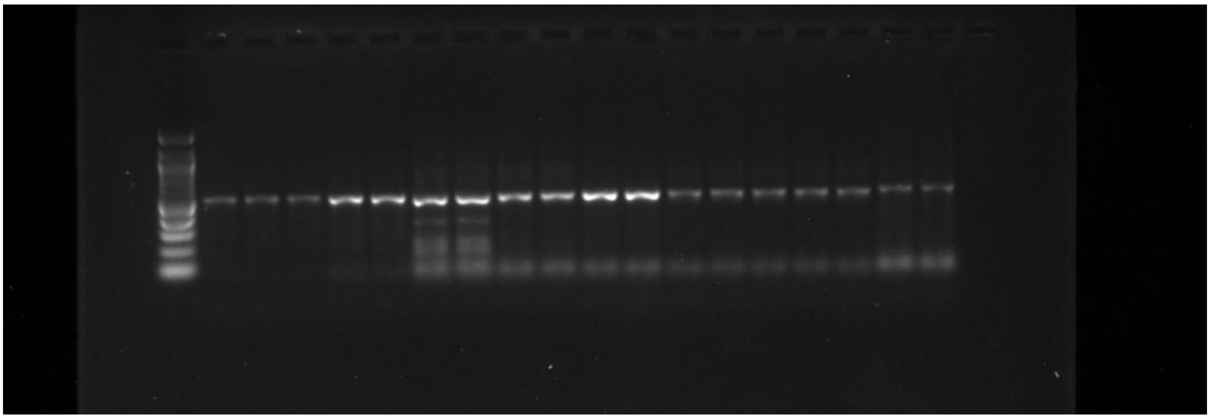

Fig 4: Agarose gel electrophoresis depicting *Salmonella ant* resistance gene from confirmed isolates.

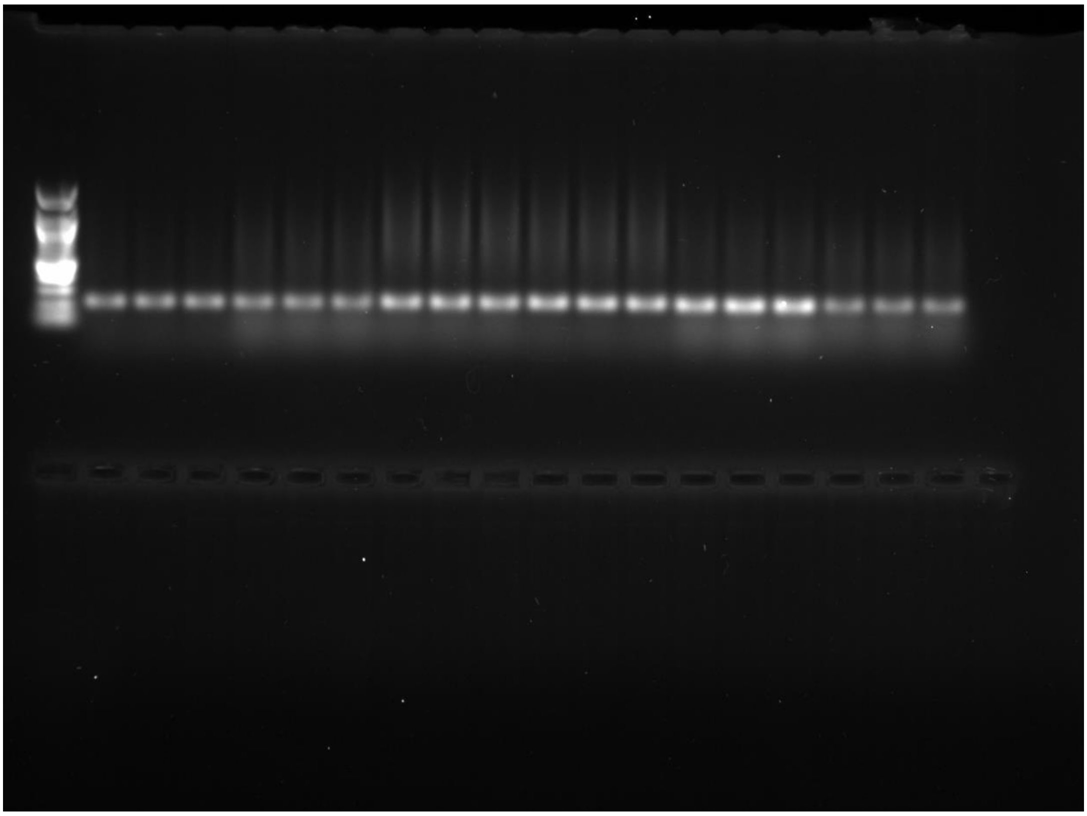

Fig 5: Agarose gel electrophoresis depicting *Salmonella tet* (A) resistance gene from confirmed isolates.

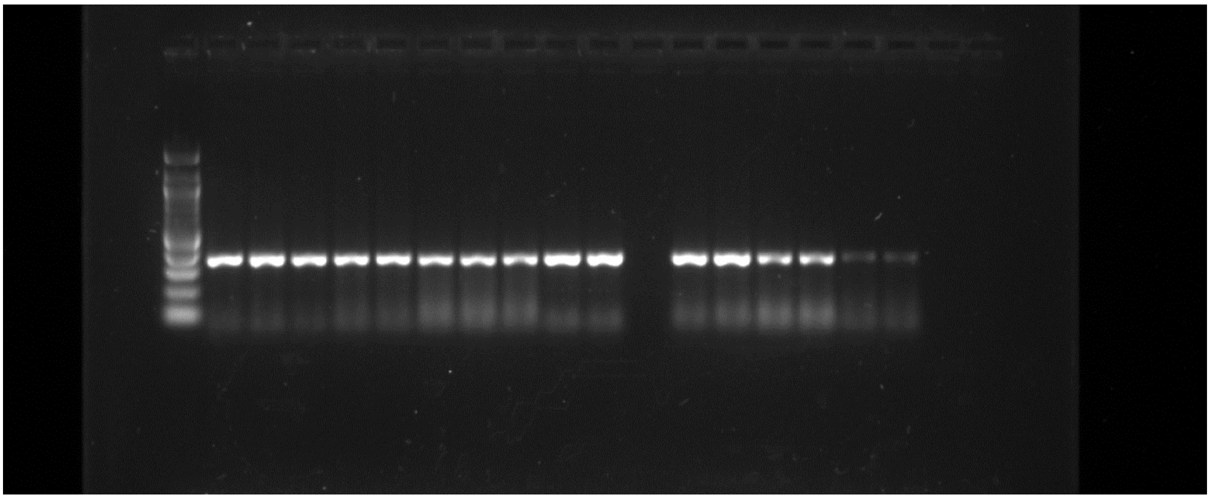

Fig 6: Agarose gel electrophoresis depicting S*almonella sui*1resistance gene from confirmed isolates.

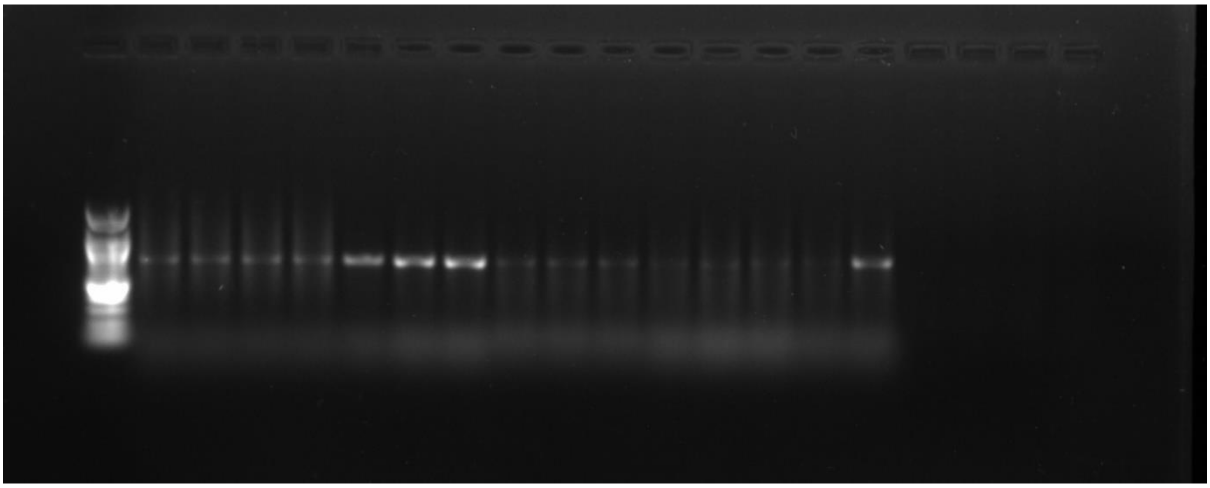

Fig 7: Agarose gel electrophoresis depicting *Salmonella sui2* resistance gene from confirmed isolates.

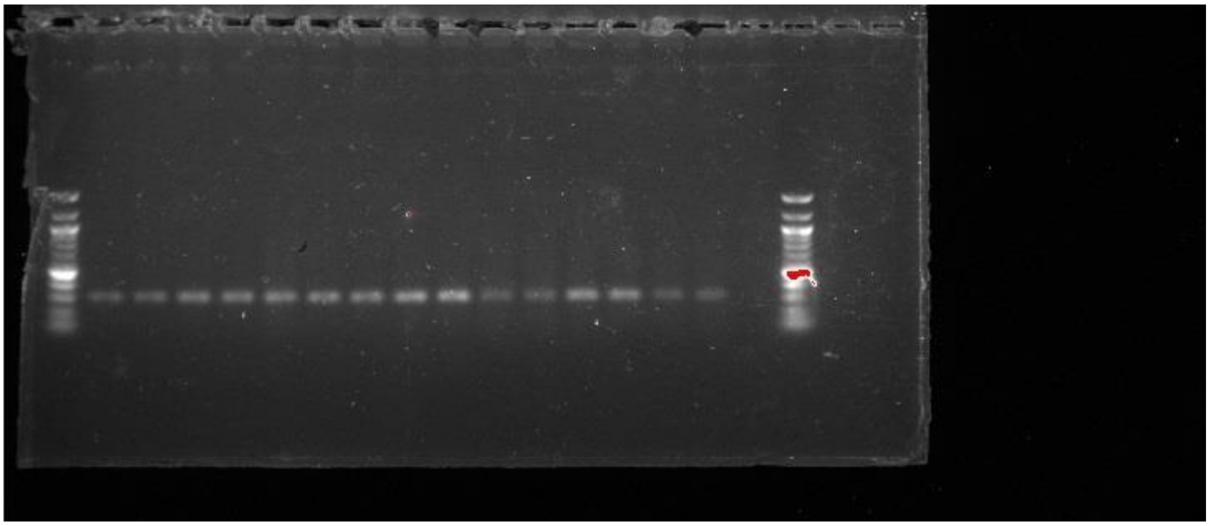

Fig 8: Agarose gel electrophoresis depicting *Salmonella spiC* virulent gene from confirmed isolates.

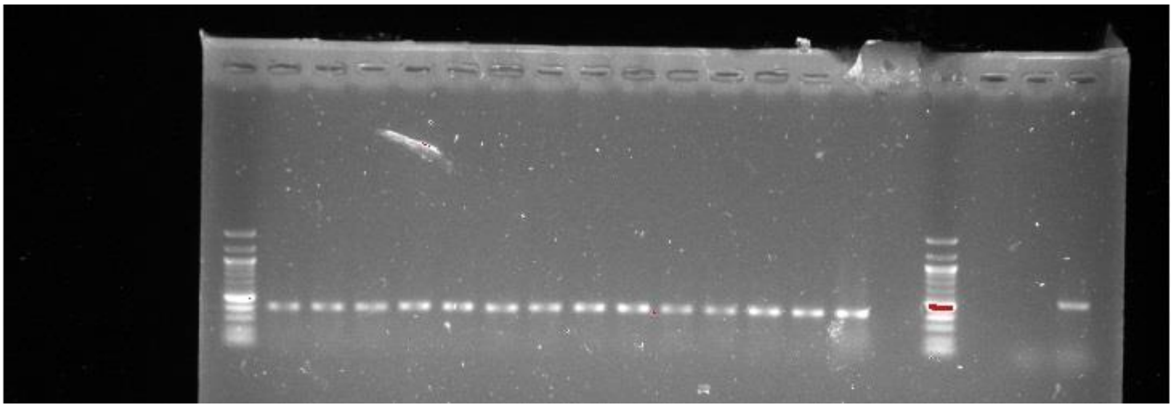

Fig 9: Agarose gel electrophoresis depicting *Salmonella misL* virulent gene from confirmed isolates.

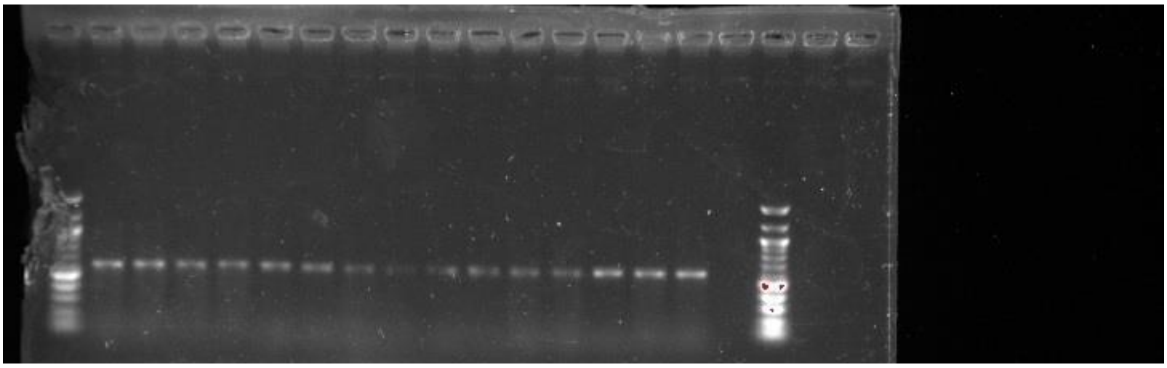

Fig 10: Agarose gel electrophoresis depicting *Salmonella orfL* virulent gene from confirmed isolates.
