# Supplementary material for: Virulence, multiple drug resistance, and biofilm-formation in Salmonella species isolated from layer, broiler, and dual-purpose indigenous chickens
Source: PLoS One. 2024 Oct 28;19(10):e0310010. doi: 10.1371/journal.pone.0310010 (PMC11515961; doi:10.1371/journal.pone.0310010)
Supplement: Attachment — Submitted filename: Response to Reviewers.docx [file pone.0310010.s004.docx]

**Reviewer #1:**

**Comment:** The manuscript contains numerous errors in grammar, punctuation, and sentence structure. The author may seek assistance from a scientific writer or revise the manuscript themselves, ensuring all necessary corrections are made before submission to another journal. **Response:** Thank you for the valuable comments. We attended to these language use issues as suggested.

**Comment:** L33: “S. Typhimurium” – only “S.” must be italics, without to italicize Typhimurium

**Response:** *S. Typhimurium* was changed to *S*. typhimurium

**Comment:** L53: “antimicrobials” instead of “antibiotics”, is a more appropriate term
**Response:** the term ‘antibiotics’ was changed to ‘antimicrobials’ as suggested.

**Comment:** L57 “enteritidis” – with sentence case

**Response:** ‘enteritidis’ was changed as suggested.

**Comment:** L87: the sampling strategy and design are unclear. How many samples were collected? The frequency of the collected samples and their amount are undefined. Was any sample size calculation taken into consideration? Information in farming systems are not described.

**Response:** The following information has been added: For microbiological analysis the minimum sample size of 75 was determined to be adequate for the study using a previously reported formular [22]. To achieve this, 5 samples were collected in each farm, directly from the cloaca of five randomly selected individual birds using sterile swabs containing multipurpose universal transport medium (line 127-129)

The farming systems have now been described (Lines 119-126).

**Comment:** L116: the importance of the screening of InvA, fliC, and Prot6e genes (why the authors chose these genes) in molecular identification and confirmation of *Salmonella* isolates are not mentioned

**Response:** The importance of screening the above indicated genes has been explained as follows: ‘This set of primers have been previously used to confirm the identity of the genus *Salmonella* and distinguish between *S*. typhimurium and *S*. enteriditis strains from other *Salmonella* serotypes [25]’. (Lines 162-165)

**Comment:** L138: the authors not mentioned the using of positive controls within the PCR reactions in order to evidence the virulent genes. Therefore, the obtained results cannot be validated!

**Response:** The use of negative control eliminates false positive results. In addition, the electrophoresis gel pictures showed that all the amplicons reflected the expected size of the targeted genes.

**Comment:** L150: the number of the tested antimicrobials is limited. It would be useful to present the classes they belong to

**Response:** The classes of antimicrobials to which the selected antibiotics belong have now been added (Lines 197-199).

**Comment:** L166: the definition for multiple antibiotic resistance is wrong “multiple antibiotic resistance (MAR) phenotypes were generated for isolates that were resistant to three or more antimicrobial agents [26]”. Therefore, the accounted results are mistakenly presented.

**Response:** The definition has been revised to read: ‘Antibiotic resistance (MAR) phenotypes were generated for isolates that were resistant to two or more antimicrobial agents’ (Lines 209-211)

**Comment:** It is also unclear, why the authors used authors E. coli as positive control in testing antimicrobial susceptibility profile of *Salmonella* strains. (All explained)

**Response:** *Escherichia coli* ATCC 25922 was used as a reference strain because it is a recommended strain for antimicrobial susceptibility test and its quality control guidelines permit greater accuracy in interpreting AMR results [29,30]. (Lines 207-209)

**Reviewer #2:**

**Comment:** This study shows a hot issue that an important bacterial contaminated specie via the food chain such as *Salmonella* and also mentioned its antibiotic resistance regarding a public health concern. Although, the study is interesting, the manuscript is lacking some important information as follows:

**Response:** We are grateful for your valuable comments. We have responded to the comments below.

**INTRODUCTION**

**Comment:** -Page 4: lines 76-78: Mention *Salmonella* biofilm forming in a recent situation in poultry slaughter houses and prove it with reference (s). The biofilm-forming *Salmonella* and its antibiotic resistance status should also be mentioned.

**Response:** The following information has been provided: Multi-drug resistant *Salmonella enterica* with the ability to form biofilm has been reported in processing equipment’s in poultry facilities [20], suggesting increased risks for recurring contamination of poultry products. The potential of pathogenic bacteria including *Salmonella* species to form biofilms affects food safety even for products preserved in appropriate storage and refrigerated temperatures [21] (Lines 88-93)

**MATERIAL AND METHODS
Comment:** Page 4: line 88: “A total of 15 poultry farms…” Please provide total birds and the number of birds sampled from each farm.

**Response:** The following information has been provided: A total of 75 faecal samples were collected from 15 poultry farms (5 layers, 5 broilers, and 5 indigenous dual-purpose chickens). The faecal samples were collected directly from the cloaca of five randomly selected individual birds per farm, using sterile swabs containing multipurpose universal transport medium. (Lines 131-135)

**RESULTS
Comment:**-Page 4, lines 210-214: “Broiler chicken flocks (average size: 5000 birds)…, most of which had foot baths for biosecurity. Layer flocks... Indigenous chicken flocks… no biosecurity measures in place.” These are housing and hygienic background in each farm and chicken type. Information are not the results. These sentences should be moved to Materials and Methods section.

**Response:** The correction has been done as suggested. (line 119-126)

**Comment:** Page 14, line 262: “Phenotype Ac1…Ac2…” Please, give the full name for Ac and then abbreviated.

**Responses:** All observed phenotypes were given a specific antibiotypes codes (Ac) with a distinct number to differentiate between biotypes and they ranged between Ac1 and Ac20 (Lines 302-305)

**DISCUSSION**

**Comment:** Page 22-24: Discussion on more interesting topics should be added, including the relationship between phenotypic and genotypic antibiotic resistance (AMR), biofilm formation, and virulence genes discovered in *Salmonella* and their AMR characteristics.

**Response:** Discussions on the above suggested topics have been added. Lines 461 – 471 and Lines 516 – 524.

**Comment:** Page 15: As the result of MAR index values, discussion should be mentioned how important of the values are?

**Response:** The discussion on the importance of the MAR index values has been included (line 444-447)

**Reviewer #3:**

Abstract

**Comment:** Line 23-26: The Background statement should be collapsed into one sentence

**Response:** The background statement has now been condensed into one sentence as follows: ‘Globally, the significant risk to food safety and public health posed by antimicrobial-resistant foodborne *Salmonella* pathogens is driven by the utilization of in-feed antibiotics, with variations in usage across poultry production systems.’ (line 23-25)

**Comment:** Line 35-36: Not comprehensible. State the predominant patterns

**Response:** The dominant antibiotic resistance phenotypes were: SXT-W-TE (16%), E-W-TE (10%), AML-E-TE (10%), E-SXT-W-TE (13%), and AMP-AML-E-SXT-W-TE (10%). (line 34-36).

**INTRODUCTION**

**Comment:** Line 80: Something is missing in “safety food products…”

**Response:** Revised to **‘**safety of food products’

**Comment:** The justification for the study seems insufficient. One is expected to understand whether studies on *Salmonella* in poultry have previously been conducted in the study area and the gap in knowledge

**Response:** The following has been added to the justification section: ‘Several studies conducted on *Salmonella* species in poultry from study area focused on the prevalence of virulent and AMR *Salmonella* serovars [10;11;12], with no consideration of the influence of poultry production systems and management practices.’ (line 71 – 73)

**Comment:** Line 81 and 84: biofilm forming should be one word “biofilm-forming”

**Response:** Revised accordingly.

**MATERIALS AND METHODS**

**Comment:** Line 102 and 106: Check the correct way of writing Salmonella shigella agar

**Response: ‘**Salmonella shigella agar’ was revised to ‘Salmonella Shigella Agar (SSA)’

**Comment:** Line 136, 138, 140, 228, table 4: change virulent genes to virulence genes
**Response: ‘**Virulent genes’ was revised to ‘virulence genes’

**Comment:** Table 2: There is no need for the rows on mechanism of resistance and broad action –

**Response:** Rows on mechanism of resistance and broad action in Table 2 was removed.

**Comment:** Line 152 -155: The choice of antibiotics for susceptibility testing should be based on standard recommendation by CLSI, EUCAST or any other organization. Testing the effect of an antibiotic against an organism that the drug is not indicated for its treatment, is not recommended because the need for the test is to guide therapy.

**Response:** The statement was revised as follows: ‘The choice of selected antibiotics was based on the standard recommendation by CLSI, particularly antibiotics that are commonly used in the treatment of bacterial infections in both humans and animals.’ (line 191-193)

**Comment:** Line 159: recommended by which organization (CLSI, EUCAST?). There is need to follow standard procedure for reproducibility. The reference [23] in line 162 is not adequate in classifying the isolates as resistant or otherwise. It is recommended that CLSI in reference [26] criteria be used in classifying the isolates

**Response:** The sentence has been revised to indicate that the recommendation was by CLSI organization. The CLSI in reference [30] (previously reference [26]) criteria was used to classify the isolates. (line 205-207)

**Comment:** Line 169-171, 430-432: Check the correct way of writing the resistance genes

**Response:** The resistance genes were revised to *sul1* and *sul2*

**Comment:** Line 91-92: There is need for the questionnaire used in assessing poultry farm husbandry practices to be referenced as a supplementary material. The survey needs to be detailed; for example, how were the questions generated, what are the themes addressed by the questionnaire, how was the questionnaire pretested for reliability and validity, administered to avoid the farmers/respondents giving socially desirable answers, and the data analyzed. These are important for reproducibility.

**Response:** The questionnaire has now been referenced as a supplementary material. (line 107-108)

To provide additional detail regarding the survey, the following information has been provided: ‘Prior to sample collection 15 (5 broilers, 5 layers and 5 dual-purpose indigenous chickens) poultry farms were identified and selected. The owners of the farms were approached to participate in the study based on willingness. Data on antibiotic treatment history and related husbandry practices were collected from 15 poultry farms (5 broilers, 5 layers and 5 dual-purpose indigenous chickens) through a structured questionnaire (S1 Survey). The questionnaire elicited information related to the poultry farmers’ demographic, husbandry practices, antibiotic use, and their knowledge of *Salmonella* spp. The survey instrument was face and content validated prior to administration by experts in the field of study. The reliability test of the instrument was carried out through the test-re-test reliability procedure by administering the questionnaire to two (2) poultry farmers at an interval of one week. The responses from the two administrations were then correlated and a high correlation coefficient of r = 0.80 was obtained. This confirmed the consistency and reliability of the instrument. Furthermore, a multiple contact strategy was used to eliminate sampling error and to ensure accuracy of responses gathered from the farmers. These eliminated the risks of receiving socially desirable responses from the farmers.’ (line 102-118)

**Comment:** Table 3: There may be no need for row on the mechanism of resistance

**Response:** The row on the mechanism of resistance was removed from Table 3 which is now table 2

**Comment:** Line 182 to 183: The statement “Pseudomonas is a strong biofilm former” needs to be clarified. Does it mean that all Pseudomonas isolates/species are biofilm-producers? If no, for reproducibility, there is need to state the type culture of Pseudomonas strain used

**Response:** We have now indicated that it is *Pseudomonas aeruginosa* ATCC 27853 that is being referred to.

**Comment:** Line 198 and Line 206: What is the meaning of SAS and LSMEANS, respectively?

**Responses:** These acronyms have now been written in full as: Statistical Analysis System (SAS) and Least squares means (LSMEANS)

**Comment:** Line 197-206: Statistical tool and data analysis only focused on optical density/biofilm formation. How were the other generated data analyzed? There would be need to test for statistical significance between the prevalence of *Salmonella* in the different chicken types since the prevalence results were compared/discussed extensively

**Response:** Agreed. We have now analysed the remaining data and added the following information to the revised manuscript: **‘**Proportional data (arising from discrete counts) were analysed using the multinomial logistic regression procedure of SAS (2010). In the categorical variable 'bird type,' the reference category selected was broiler due to the reported extensive use of antibiotic growth promoters in this group. Consequently, there was an anticipation of a higher likelihood of detecting antimicrobial resistance in broilers.’(line 252-256)

**RESULT**

**Comment:** Line 219-220: Repetition of the methodology in line 117-118

**Responses:** The repetition was removed.

**Comment:** Line 220-221: Recast as thus: “Out of 150 isolates, 82 (55%) were confirmed to be *Salmonella*”

**Responses:** Revised as suggested. (line 259)

**Comment:** Table 4: The title could be “Proportion of isolates positive for…”. Then the row with bird type should be “Bird type (%)” while the top will be “Percentage of isolates positive for specie-specific gene”.

**Responses:** Thank you for these suggestions. We have modified the table caption accordingly.

**Comment:** Moreover, are invA, fliC and prot6e virulence genes? If yes, why were they separated from others-

**Responses:** Thank you for this comment; The genes are virulent genes, particularly *invA* gene, hence the tables have now been merged into one. Despite the above, virulent genes are sometimes used as biomarkers to confirm the identity of isolates or *Salmonella* serovars. This is because the *invA* gene contains sequences unique to the genus *Salmonella* while *fliC* and *prot6e* contain sequences unique to *S.* typhimurium and *S.* enteritidis, respectively and they have been previously used as biomarkers for Salmonella genus and serovars [25].

**Comment:** Lie 254,411: gentamycin is incorrect

**Response: ‘**gentamycin’ was corrected to ‘gentamicin’

**Comment:** Table 5: The presentation is not comprehensible. For example, what does the figures represent? I assume it is the concentration of the antibiotics in the discs. Therefore, these concentrations should have been mentioned in the methodology.

**Response:** The concentration of the antibiotics were mentioned in the methodology as follows: The list of used antibiotics comprised of gentamicin (GM10 μg), amoxicillin (A10 μg), erythromycin (E15 μg), chloramphenicol (C30 μg), tetracycline (T10 μg), trimethoprim (TM25 μg), ampicillin (AP30 μg), trimethoprim-sulfamethoxazole (TS25 μg), and kanamycin (K30 μg) [26, ] (line 193-197)

**Comment:** The title should also be rephrased as mentioned earlier

**Response:** The title has now been revised to read: Proportion of isolates resistant to tested antibiotics.

**Comment:** Line 273-277: check the proper way of writing the genes

**Response:** All genes were corrected as follows: ‘*ant (3”)-la*, *sui1*, *sui2*, *tet* (A), and *tet* (B)’

**Comment:** Table 7: There were two table 7 containing different information. The table 7 on biofilm is not necessary, it can be described

**Response:** The table 7 on biofilm has been removed and results described as suggested. (line 369-379)

**Comment:** The gel pictures were not fully labelled. For example, which lane represents the marker and the isolates?

**Response:** All gel pictures captions are included in the manuscript as stipulated in the journal’s guidelines

**Comment:** Discussion Line 340-354, 397-403: Unnecessary

**Response:** The opening paragraph under discussion has now been removed.

**Comment:** Line 356-363: Did not actually discuss the result of the study
**Response:** The subtopic poultry husbandry practices has now been moved to methodology section upon reviewer #2’s suggestion. (line 119-126)

**Comment:** Line 367-368: The comparison with references 6 and 36 seems inappropriate
**Response:** The comparison statement between the two references (6 and 36) has now been removed.

**Comment:** Line 377: Did reference 38 and 39 compared *Salmonella* prevalence in the different chicken types?

**Response:** Yes, one of the studies compared *Salmonella* prevalence in broilers, layers, and indigenous chickens across different seasons while the other only compared *Salmonella* prevalence in broilers chickens and free range.

**Comment:** Line 399: Italicize *Salmonella*

**Response:** *Salmonella* was italicized

**Comment:** Line 40-402: This statement “In the current study, *Salmonella* isolates were tested against 9 different antibiotics and results revealed that” is unnecessary.

**Response:** The statement has been deleted as suggested.

**Comment:** Line 396-446: The resistance gens should be related to the phenotypes. If antimicrobial use was assessed by the questionnaire, it would have been easier to back-up the use of the antibiotics as the cause of the resistance.

**Response:** The resistance genes have now been correlated with the phenotypes data in the discussion section (line 462-472).

The findings of the study revealed that *Salmonella* isolates phenotypic and genotypic resistance patterns were not influenced (p > 0.05) by bird type. Despite the above, the survey results showed that antimicrobial use differed significantly across the different bird types.

**Comment:** The authors detected resistance genes in isolates from indigenous chickens whereas antibiotics are not used in raising these birds. It is expected that the possible source(s) of the genes be postulated.

**Response:** The possible sources of the resistance genes in indigenous chickens has been now discussed (line 432-441)

**Comment:** Line 466-467: What is the implication of the finding that “Most isolates were strong biofilm formers, regardless of incubation temperature”

**Responses:** The implication of the finding “Most isolates were strong biofilm formers, regardless of incubation temperature” has been explained (line 520-526).

**CONCLUSION**

**Comment:** The conclusion did satisfactorily answer the objective of the study. For example, it did not capture the husbandry practices and prevalence/occurrence of *Salmonella* in the different production systems and the implication of finding resistance and virulence genes in *Salmonella* isolates from domesticated and indigenous poultry birds

**Response**: The conclusion has been now revised to answer the main question of the study and explain the implications of detecting AMR pathogenic *Salmonella* isolates in different bird types (line 530-539)

**Response**: The limitation of the study was the number of participating poultry farms which also affected the sample size.
