# Supplementary material for: Virulence, multiple drug resistance, and biofilm-formation in Salmonella species isolated from layer, broiler, and dual-purpose indigenous chickens
Source: PLoS One. 2024 Oct 28;19(10):e0310010. doi: 10.1371/journal.pone.0310010 (PMC11515961; doi:10.1371/journal.pone.0310010)
Supplement: Attachment — Submitted filename: Response to Reviewers.docx [file pone.0310010.s005.docx]

**Reviewer #1:** Even though the authors tried to address the concerns raised by the reviewer, the wording below, in response to a critical concern, compromises the results of the study.

**Previous reviewer comment:
Comment:** L166: the definition for multiple antibiotic resistance is wrong “multiple
antibiotic resistance (MAR) phenotypes were generated for isolates that were resistant
to three or more antimicrobial agents [26]”. Therefore, the accounted results are
mistakenly presented.

**Author response in revision**
"Response: The definition has been revised to read: ‘Antibiotic resistance (MAR)
phenotypes were generated for isolates that were resistant to two or more antimicrobial
agents’ (Lines 209-211)"

This statement is scientifically invalid. Please consult: doi: 10.1111/j.1469-0691.2011.03570.x.
Magiorakos et al., 2012.

**Response:** Thank you for this comment, the statement has been now revised to: “multiple antibiotic resistance (MAR) phenotypes were generated for isolates that were resistant to at least one agent in three or more antimicrobial categories’’. [Lines 206-209]

**Reviewer #2:** Revision has improved and provided more important aspects of the manuscript. However, some concerns are as follows:

**Introduction:**

-Page 4: lines 92: Prevalence of biofilm-forming Salmonella and its antimicrobial resistance in poultry farms in your country or other countries in previous or recently studies should also be mentioned.

**Response:** Revised as follows: Recent studies have reported a high prevalence of multi-drug resistant biofilm-forming *Salmonella* serovars in poultry farms and processing facilities globally [20,21,22,23,24], suggesting increased risks for recurring contamination of poultry products. [Lines 88-91]

**Discussion**

**Comment**
-Page 23/ line 440: The sentence, “…MDR to more than two tested antibiotics…” should be changed to “…MDR to more than two tested antibiotic classes…” because MDR means bacteria resisted to more than two tested antibiotic classes not only resisted to the antibiotic types.

**Response:** Thank you for this comment and the statement has been revised to: “MDR to three or more tested antibiotics classes”. [Line 454]

**Other**

**Comments**
Table 2: “…of resistant genes in Salmonella isolates”. The phrase is not complete, should be changed to “…of resistant antimicrobial genes in Salmonella isolates”.

**Response:** The phrase has been revised to: “of resistant antimicrobial genes in *Salmonella* isolates”. [Table 2]

**Comment**
Table 3,4,6: The data (genus, specie, and virulence genes or resistant isolates) have presented as the percentage. However, the number of resulted isolates are not clear, so the authors should provide the number of isolates (maybe in parenthesis) for each observed value.

**Response:** Thank you for the comment, the total number of isolates **(N)** are indicated in table 3,4,6, to make it easier for readers to calculate the number of isolates without cluttering the tables by presenting both the number and percentage in each cell.

**Comment**
Table 3,4,6: Please, express the statistical analysis in the tables along with the table legends.

**Response:** The statistical analysis has now been stated in the table footnotes (Table 3,4,6) as follows: “Proportional data were analysed using the multinomial logistic regression procedure of SAS (2010)”

**Comment**
All figures (gel pictures): The author should provide the company and country of DNA maker used.

**Response:** The company and country of DNA maker used have now been provided in all gel picture legends as follows: **“**Thermo Fisher Scientific, South Africa”
