# Supplementary material for: Virulence, multiple drug resistance, and biofilm-formation in Salmonella species isolated from layer, broiler, and dual-purpose indigenous chickens
Source: PLoS One. 2024 Oct 28;19(10):e0310010. doi: 10.1371/journal.pone.0310010 (PMC11515961; doi:10.1371/journal.pone.0310010)
Supplement: Attachment — Submitted filename: Response to Reviewers.docx [file pone.0310010.s006.docx]

**Reviewer #3:**

**Comment:** 39: Give space theSPI-3

**Response:** theSPI-3 was revised to ‘the SPI-3’

**Comment:** 102: Put a comma after “Prior to sample collection”

**Response:** Done as suggested.

**Comment:** 141: Write “Salmonella Shigella Agar” as Salmonella-Shigella agar and italicize Salmonella and Shigella

**Response:** “Salmonella Shigella Agar” was revised as advised to ‘*Salmonella-Shigella* agar’

**Comment:** 142: Change “Lactose-negative” to Lactose non-fermenting

**Response:** “Lactose-negative” was revised to ‘Lactose non-fermenting’

**Comment:** 144: Change “lactose-positive” to “lactose-fermenting”

**Response:** “lactose-positive” was revised to ‘lactose-fermenting’

**Comment:** 305: This statement should not be under table 4 as the table did not show any data analyzed with the tool “Proportional data were analysed using the multinomial logistic regression procedure of SAS (2010)” Further, it could be observed that all the isolates from broilers and layers were reported as susceptible to gentamicin – this report is contrary to the cited CLSI document which stated that Salmonella isolates should always be reported as resistant to gentamicin and streptomycin because these aminoglycosides although might show in vitro activity but are not utilized clinically.

**Response:** Thank you for this comment, the statement was removed under table 4. Thank you for this observation and we do agree with you. To clarify the above, the following statement was added: “Despite that aminoglycosides such as gentamicin and kanamycin displayed *in-vitro* activity against the *Salmonella* isolates in this study, these isolates should be considered as resistant based on the CLSI recommendation, since these drugs are not used clinically.” However, there is evidence of a report indicating that *Salmonella* infections caused by isolates that are resistant to first line antibiotics may be treated with alternative second line antimicrobial agents such as aminoglycosides principally amikacin, or folic acid pathway inhibitors like sulfisoxazole or sulfamethoxazole with or without trimethoprim as well as carbapenems, imipenem or meropenem which are administered intravenously (Guerrant *et al.,* 2001).

**Comment:** 320-325: Correct “sui1, sui2,” They should be sul1, sul2 and italicized. Also correct them in line 494 and table 6

**Response:** Done as suggested.

**Comment:** 483: Check tet (B)
S, streptomycin appeared as a legend underneath table 5 whereas there was no resistance to streptomycin

**Response:** S, streptomycin was removed as a legend underneath table 5 and tet (B) was fixed

**Comment:** 475-476: Give examples of the silent genes observed in your experiment

**Response:** Thank you for this comment and the silent genes were added as follows; particularly tet (A), tet (B) and *ant (3”)-la*

**Comment:** 486: resistance not resistant

**Response: ‘**Resistant’ was revised to ‘resistance’

**Comment:** 533: Put a comma after “In conclusion”

**Response:** Done as suggested.
